# Hematological Indices for Identifying Adverse Outcomes in Children Admitted to Pediatric ICUs

**DOI:** 10.7759/cureus.53744

**Published:** 2024-02-06

**Authors:** Sivakumar Mahalingam, Vikram Bhaskar, Prerna Batra, Pooja Dewan, Priyanka Gogoi

**Affiliations:** 1 Pediatrics, University College of Medical Sciences, Delhi, IND; 2 Pediatrics, University College of Medical sciences, Delhi, IND; 3 Transfusion Medicine, All India Institute of Medical Sciences, Guwahati, Guwahati, IND

**Keywords:** pediatric intensive care unit (picu), neutrophil to lymphocyte ratio (nlr), platelet to lymphocyte ratio (plr), pediatric mortality, hematological indices

## Abstract

Background: The pediatric ICU (PICU) is a specialized area where critically sick children are managed. The mortality rates in PICUs are higher in developing countries as compared to developed nations. Many of these deaths could be prevented if very sick children were identified soon after they arrived at the health facility. Hematological indices like platelet lymphocyte ratio (PLR) and neutrophil-lymphocyte ratio (NLR) have been frequently used in adults as indicators of mortality. However, their use in the pediatric population is limited due to a lack of validated reference intervals.

Objective: The objective of the study is to assess the role of hematological indices in identifying adverse outcomes in terms of mortality in children admitted to the PICU.

Materials and methods: It is a prospective, observational study done at a tertiary care hospital. All children aged one year to 12 years admitted to the PICU were enrolled in the study. A sample for complete blood count was taken within one hour of admission to the PICU. Children who had received blood products in the last two months, those on chronic medications (>two weeks) that can affect bone marrow cellularity, and known cases of hematological disorders such as megaloblastic anemia, hematological malignancies, immune thrombocytopenia, and aplastic anemia were excluded from the study. PLR, NLR, and platelets to mean platelet volume ratio (PLT/MPV) were determined and compared among the survivors and non-survivors.

Results: Out of 275 enrolled patients, 119 (43.3%) patients expired during the study period. While PLR had high sensitivity and NLR had high specificity (85.71% and 92.31%, respectively) for predicting mortality, none of these parameters had a good area under the curve (AUC) in our study. PLT/MPV of ≥32 had a sensitivity of 39.5% and a specificity of 56.41% for predicting mortality.

Conclusions: Hematological parameters have been used across the world to predict ICU mortality. PLR and NLR are simple hematological biomarkers, easy to calculate, and cost-effective, and ratios are better than individual parameters. More studies and stratified samples are required to evaluate the role of hematological markers in identifying the risk of mortality in children admitted to PICUs.

## Introduction

The pediatric ICU (PICU) is a specialized area where critically sick children are managed. The mortality rates in PICUs can be as high as 40-58% in developing countries [1,2]. Many of these deaths can be prevented if very sick children are identified soon after their arrival in the health facility, and treatment is started immediately [3]. Several biomarkers like C-reactive protein (CRP) [4], red cell distribution width (RDW) [4,5], and procalcitonin [6], etc. have been used as predictors of mortality and critical illness in patients admitted to PICUs. The availability and cost of these biomarkers are the main limiting factors, especially in developing countries. Various clinical scores like Pediatric Logistic Organ Dysfunction 2 (PELOD 2), Pediatric Risk Mortality 3 (PRISM 3) [7], and Sequential Organ Failure Assessment (SOFA) [8] are also in use, which require multiple parameters to predict mortality. These scores have been designed to augment the clinical acumen and provide an objective basis to ascertain the risk of mortality; however, they need several laboratory investigations (serum creatinine, aspartate transaminase, prothrombin time, arterial blood gas analysis serum electrolytes, etc.), which add to the cost of treatment and are often complicated to perform.

A complete blood count (CBC) is a basic investigation, which is easily available at most centers. Whenever the body mounts an inflammatory response to any stress or infection, the composition of white blood cells, platelets, and other components in blood changes.

Platelet to lymphocyte ratio (PLR) has been identified as a novel, independent prognostic marker of outcome. The prognostic value of PLR has been studied in critically ill patients with acute kidney injury by Zheng et al. [9]. They observed that both low and high PLRs were associated with increased mortality. PLR has been found to be useful in different clinical settings that include pneumonias, infections, and trauma [10,11].

In response to systemic inflammation or stress, there is an increase in neutrophil production and a decline in lymphocytes, resulting in an increase in neutrophil to lymphocyte ratio (NLR). It has been found that NLR can be a surrogate marker for endothelial dysfunction and inflammation [12,13]. Critically ill patients also show some changes in mean platelet volume (MPV) apart from platelet counts. MPV reflects the size of the platelets. Young platelets are larger than older platelets. Increased number of young platelets indicates increased platelets production due to overconsumption induced by inflammation. Larger platelets are functionally, metabolically, and enzymatically more active and thus MPV is considered as a better marker of platelet function than the platelet count itself. The aim of the present study was to assess these hematological indices for identifying mortality in children admitted to the PICU.

## Materials and methods

This prospective observational study was conducted at the University College of Medical Sciences, Delhi, India, from January 2021 to August 2022 after obtaining ethical clearance from the Institutional Ethical Committee (Approval No. IEC-HR/2020/PG/46/74, dated Dec 21, 2020). Written informed consent was taken from parents/guardians. A total of 275 children aged one year to 12 years, admitted to the Pediatric Intensive Care Unit, were enrolled in the study. Children who had received blood products in the last two months, children who were on chronic medications (>two weeks) that can affect bone marrow cellularity, and known cases of hematological disorders such as megaloblastic anemia, hematological malignancies, immune thrombocytopenia, and aplastic anemia were excluded from the study. Patients' complete demographic details, chief complaints, anthropometric parameters, vital signs at admission including temperature, pulse rate/heart rate, respiratory rate, oxygen saturation (SPO2), blood pressure (systolic, diastolic, and mean), Glasgow Coma Scale (GCS), pupillary reaction, PELOD-2 score, investigations including CBC, serum lactate, arterial blood gas parameters, liver function tests, and kidney function tests were noted. The provisional diagnosis, final diagnosis, and final outcome were recorded in a predesigned proforma.

The primary objective was to assess the performance of PLR ≥ 3.9, NLR ≥ 7, and PLT/MPV ≥ 32 for identifying adverse outcomes [14], namely mortality, prolonged PICU stay, and prolonged hospital stay in children admitted to the PICU. A total of 2 mL of venous blood was collected from the peripheral accessible veins in an ethylenediamine tetraacetic acid (EDTA) vial within one hour of admission to the PICU. Complete blood indices were assessed by an automated hematology analyzer and ratios (PLR, PLT/MPV, and NLR) were subsequently calculated from the values obtained from the analyzer using the following formulas: 1. Platelet to lymphocyte ratio (PLR) = (absolute platelet count)/(absolute lymphocyte count). 2. Total platelet count to mean platelet volume (PLT/MPV) = (total platelet count)/(mean platelet volume). 3. Neutrophils to lymphocytes ratio (NLR) = (percentage of neutrophil count)/(percentage of lymphocyte count).

Sample size

In a study Misirlioglu, et al. [14] observed that PLR ≥ 3.9 at admission can determine all-cause mortality in pediatric trauma patients with 80% sensitivity. Taking 10% absolute precision on both sides and a 95% confidence level, the sample size was calculated to be 62. Data from our PICU shows a mortality of 25% among children admitted to PICU. To get the sample size of 62, we needed a total of 248 children. Considering attrition due to loss to follow-up and sample processing errors, we enrolled 275 children in our study.

Statistical analysis 

Data were analyzed and statistically evaluated using IBM SPSS Statistics for Windows, Version 25, (Released 2017; IBM Corp., Armonk, New York, United States). The normal distribution of different parameters was tested by the Shapiro-Wilk normality test. Normality differences between the mean of the two groups (survivors vs. non-survivors) were compared by the student t-test or Mann-Whitney U test. Statistical differences between the proportions were tested by the chi-square test or Fisher’s exact test. Based on the area under the curve (AUC), the cut-off value was calculated using the Youden Index, and sensitivity, specificity, positive predictive value (PPV), and negative predictive value (NPV) of PLR, PLT/MPV, and NLR were calculated. A P-value <0.05 was considered statistically significant.

## Results

One hundred and nineteen (43.3%) enrolled patients expired during the study period. No significant correlation was found between age group, gender, anthropometric parameters, and vital parameters with mortality in PICU (Tables 1, 2).

**Table 1:** Baseline demographic profile of patients admitted to pediatric ICU (PICU) Chi-square test for categorical data; #Mann-Whitney U test for quantitative data.

Variable	Total (n=275)	Survivors (n=156)	Non-survivors (n=119)	P-value
Age group n (%)				
1-2 years	157 (57.1%)	82 (52.6%)	75 (63.0%)	0.19
3-5 years	41 (14.9%)	27 (17.3%)	14 (11.8%)	
6-12 years	77 (28.0%)	47 (30.1%)	30 (25.2%)	
Median age	2 (1-12)	3 (1-12)	1 (1-11)	#0.19
Gender n (%)				
Male	158 (57.5%)	92 (59.0%)	66 (55.5%)	0.55
Female	117 (42.5%)	64 (41.0%)	53 (44.5%)	

**Table 2:** Comparison of clinical and laboratory parameters between survivors and non-survivors Student's t-test for mean ± SD or Mann Whitney u test for median (IQR); # Variables are median values; *P-value < 0.05 significant. SPO2: Saturation of peripheral oxygen; GCS: Glasgow Coma Scale; PELOD-2: Pediatric Logistic Organ Dysfunction-2; PaO2/FiO2: Ratio of partial pressure of oxygen in arterial blood to the fraction of inspiratory oxygen concentration; PaCO2: Partial pressure of arterial carbon dioxide; SGOT: Serum glutamic-oxaloacetic transaminase; SGPT: Serum glutamic pyruvic transaminase; ALP: Alkaline phosphatase; IQR: Interquartile range

Clinical parameters	Total (n=275)	Survivors (n=156)	Non-survivors (n=119)	P-value
Pulse rate	135±29.41	132±29.57	138.47±28.94	0.08
Respiratory rate	42±14.23	42±14.42	41.56±14.05	0.86
SPO2	96±6.48	96±6.84	96.91±5.96	0.37
Systolic blood pressure	91±16.67	92±14.79	90.94±18.91	0.64
Mean arterial pressure	71±11.37	71±10.89	71.73±12.01	0.57
Shock index	1.48±0.22	1.44±0.41	1.55±0.44	0.03*
Modified shock index	1.91±0.54	1.87±0.50	1.97±0.59	0.14
Median GCS	10 (6-15)	14 (10-15)	7 (5-10)	<0.001*
Median PELOD 2 score	4 (2-6)	2 (1.25-4.0)	6 (4-8)	<0.001*
Laboratory parameters				
PaO2/FiO2 ratio <61	136 (49.5%)	74 (47.4%)	62 (52.1%)	0.44
PaCO2 >59mm Hg	9 (3.3%)	2 (1.3%)	7 (5.9%)	0.04*
Lactate >5 mmol/L	20 (7.3%)	4 (2.6%)	16 (13.4%)	0.001*
Hemoglobin (mg/dL)	10.49±2.36	10.59±2.30	10.35±2.45	0.22
Total leucocyte counts (per mm^3)^	12203±6526.6	13862±7232.4	11719±6104.6	0.48
Platelet counts (lacs/mm^3^)	3.00±1.67	3.38±1.85	2.45±1.23	0.35
#Urea (mg/dL)	28 (20-41)	27 (20-39.75)	28 (22-45)	0.04*
#Creatinine (mg/dL)	0.6 (0.5-0.9)	0.6 (0.5-0.8)	0.8 (0.5-1.0)	<0.001*
Sodium (mEq/L)	137.66±5.72	137.42±5.49	137.97±6.01	0.42
Potassium (mEq/L)	4.56±0.80	4.51±0.79	4.63±0.82	0.22
Calcium (mg/dL)	9.11±0.77	9.15±0.84	9.05±0.66	0.25
Bicarbonate (mEq/L)	19.0 + 4.35	19.73 + 3.55	18.04 + 5.06	0.46
#SGOT (U/L)	53 (30-87)	48 (24.75-77.25)	55 (35-142)	0.01*
#SGPT (U/L)	55 (38-89)	55 (35-85)	69 (40-115)	<0.01*
#ALP (U/L)	172 (148-225)	171.5 (150-222)	173 (145-243)	0.96

Parameters like shock index (1.55±0.44), hypotension, raised serum creatinine ≥ 0.8, urea ≥ 28, SGOT ≥ 55, SGPT ≥ 69, Pco2, lactate >5mmol/L, median PELOD 2 score ≥ 6, and median GCS ≤ 7 and the proportion of patients who were mechanically ventilated between the two groups (survivors vs. non-survivors) were observed to be statistically significant as shown in Table 2.

Hematological parameters namely, hemoglobin (Hb), total leukocyte count (TLC), absolute neutrophil count (ANC), platelet (PLT) count, NLR, PLR, and PLT/MPV ratio were comparable between the two groups. It was observed that platelet lymphocyte ratio ≥3.9 had a sensitivity of 85.71 %, specificity of 14.10%, PPV of 43.22%, and NPV of 56.41% for predicting mortality. Platelet to mean platelet volume ≥ 32 had a sensitivity of 39.5%, specificity of 56.41%, and NPV of 55% for predicting mortality. Neutrophil lymphocyte ratio ≥ seven had a specificity of 92.31% and an NPV of 56.69% for predicting mortality. NLR was found to predict prolonged PICU stay with higher specificity than other parameters (Table 3).

**Table 3:** Sensitivity and specificity of PLR ≥ 3.9, PLT/MPV ≥ 32, and NLR ≥ 7 for the prediction of mortality. PLR: Platelet to lymphocyte ratio; PLT/MPV: platelet count/mean platelet volume; NLR: Neutrophil to lymphocyte ratio

	Prediction of mortality		Prediction of PICU stay > three days		Prediction of hospital stay > 10 days	
	Sensitivity	Specificity	Sensitivity	Specificity	Sensitivity	Specificity
PLR ≥ 3.9	85.7	14.1	87.8	16.9	86.9	14.7
PLT/MPV ≥32	39.5	56.4	43.2	59.6	46.4	60.2
NLR ≥7	7.7	92.3	6.5	91.2	3.6	90.6

## Discussion

In this observational study, we observed that out of 275 children enrolled, 156 (56.6%) patients survived while 119 (43.3%) expired. Further, in the subgroup of 1-2 years, 3-5 years, and 6-12 years, the mortality rates were 47.7%, 34.1%, and 38.9%, respectively.

The factors that were shown to be statistically different between survivors and non-survivors in our study were invasive mechanical ventilation, shock index, MODS (multi-organ dysfunction syndrome), hyperlactatemia, a high PELOD score (> 6), and GCS < 7. We observed that 83.2 % of mechanically ventilated patients died in our study (P-value of <0.001). Mechanical ventilation is associated with a higher risk of mortality [15], especially during treatment of less than one day in PICUs. Rusmawatiningtyas et al. [16] reported a mortality rate of 96.4% in mechanically ventilated patients (P-value < 0.001) in a low-resource setting. In an adult study, Combes et al. [17] observed that prolonged mechanical ventilation for more than 14 days was associated with higher mortality (43% vs. 33%).

We also found that the shock index was higher in non-survivors (1.55± 0.44) than in survivors (1.44 ± 0.41). Shock index was found to be associated with mortality in the study by Rousseaux et al. [18] and Bhat et al. [19]. A shock index ≥ 0.9 was also found to be correlated with hospital days and ICU days [20].

We found a high PLR ≥ 3.9 in 84 % of non-survivors, though it was not able to predict mortality even at best cut-offs (AUC of 0.49). Misirlioglu et al. [14] found high PLR ratios in non-survivor patients in their study, and the cut-off value of ≥ 3.9 predicted increased mortality with a sensitivity of 80% and specificity of 68.9%. In another study by Shimoyama et al., PLR showed a sensitivity of 62.5% and specificity of 66.7% for the prediction of mortality in patients with gastro-intestinal perforation [10]. Tekin et al. [20] in their study of pediatric trauma patients found that the PLR was raised significantly in non-survivors in comparison to survivors (PLR, 145.3±85.0 vs. 46.2±25.2, P<0.001).

In the present study, we found the NLR ≥ 7 in 7.6% of non-survivors with a sensitivity of 7.56% and specificity of 92.31%. Mathews et al. [21] observed in their study that an NLR rise of > 11 was associated with mortality with a sensitivity of 61.11% and specificity of 70.27%. With an AUC of 0.727. Shimoyama, et al. [10] demonstrated that an NLR > 13.28 is associated with mortality with a sensitivity of 62.5% and specificity of 66.7%. Tekin et al. [20] found that an NLR rise of > 2.77 was associated with mortality with a sensitivity of 70% and specificity of 77%.

Further, PLT/MPV ≥ 32 was observed in 39.5% of non-survivor patients, with a sensitivity of 39.5% and specificity of 56.41% with an AUC of 0.45 for predicting mortality. Tamelyte, et al. [22] found that the rise of PLT/MPV was useful in predicting sepsis (41.42 ± 15.86 vs. 33.45 ± 17.97; P = 0.001), while Liberski et al. [23] concluded against it. Seyhanli et al. [24] observed that PLT/MPV levels decreased (<25.13) in adult COVID-19 patients as compared to healthy individuals. Su et al. [25] investigated the prognostic significance of PLT/MPV in advanced esophagus squamous cell carcinoma and concluded that a higher PLT/MPV was an independent predictor of longer progress-free survival (157 days vs. 85 days, P <.001).

The major strength of our study is that our study has a good sample size (275), with adequate statistical power (80%). This is the only prospective study that has studied PLT/MPV as an independent marker for predicting mortality in PICU. We didn’t enroll any hematological patients, patients who have received blood products prior to enrollment, and patients with chronic drug intake that affects bone marrow cellularity, thus eliminating the bias of hematological markers. We assessed all-cause mortality, thus making the results more generalizable.

This study is limited by being a single-center study. The majority of the patients were referred to our center and have already received treatment that could alter cell lines in the blood. Being a referral center, we also received critically sick and terminally ill children that contribute to unusually high mortality. We performed the tests only at admission. A change in markers during treatment might have turned out to be a better predictor.

## Conclusions

PICU mortality varies around the globe. Hematological parameters and scores were used for predicting mortality. PLR and NLR are simple hematological biomarkers, easy to calculate, and cost-effective, and ratios are better than individual parameters. Though PLR and NLR had high sensitivity and specificity, respectively (85.71% and 92.31%, respectively) for predicting mortality, none of the parameters had good AUC in our study.
